# Fentanyl versus dexmedetomidine during awake-fibreoptic intubation

**DOI:** 10.6026/973206300210418

**Published:** 2025-03-31

**Authors:** Anirudh Jayaraj, Jamale P.B, Vishwas Manohar Joshi

**Affiliations:** 1 Department of Anesthesiology, Krishna Institute of Medical Sciences, Krishna Vishwa Vidyapeeth (Deemed to be University), Karad, Maharashtra, India

**Keywords:** Fentanyl, dexmedetomidine, intubation conditions, blood pressure, heart rate, systolic blood pressure, diastolic blood pressure

## Abstract

Awake-fiberoptic intubation is considered the gold standard technique for managing an anticipated difficult airway. Therefore, it is of interest to compare and evaluate fentanyl and dexmedetomidine on intubation conditions during awake-fiberoptic intubation. Hence, 90 patients were randomly divided into two groups, namely Group D and Group F, each consisting of 45 individuals. They were given Dexmedetomidine (1 mcg/kg over 10 minutes) and fentanyl (2 mcg/kg over 10 minutes) followed by monitoring and recording using Ramsay sedation scale at every 2 minutes, 5 minutes, 10 minutes and 20 minutes. Parameters like systolic blood pressure, diastolic blood pressure and heart rate were noted. They found that, the differences are not statistically significant as time advances following intubation, even though dexmedetomidine contributes to the maintenance of a lower systolic blood pressure. Further, the efficacy of dexmedetomidine in reducing diastolic blood pressure is more pronounced; however, following intubation, the disparities between the two groups diminish and it is not statistically significant. Thus, in comparison to fentanyl, dexmedetomidine demonstrates superior efficacy in the management of heart rate both during and immediately following intubation.

## Background:

The estimated incidence of patients experiencing a difficult airway during clinical anesthesia is reported to range from 1% to 18% [1]. Inadequate management of the airways in these patients can lead to serious complications, including hypoxemia, hypoventilation, aspiration, brain injury, or potentially fatal outcomes [2]. When patients are expected to have difficult airways, awake-fiberoptic intubation is regarded as the most effective procedure [3]. This has led to the testing of numerous medication regimens, both individually and in combination. Nevertheless, it is not ideal for awake-fiberoptic intubation since several of these medications may induce respiratory depression, which in turn might cause airway blockage [4]. Establishing a balance between optimal intubation parameters and patient comfort is crucial during the preparation for awake-fiberoptic intubation. A significant challenge associated with this procedure is achieving adequate sedation while preserving airway patency and ensuring effective ventilation [5]. In addition, dexmedetomidine, when combined with anesthetic drugs, may enhance the effects of benzodiazepines and opioids. When it comes to sedative, anxiety-reducing, pain-relieving and sympatholytic effects during awake intubation, dexmedetomidine stands out because it keeps breathing working while still having these effects. A half-life of six minutes is characteristic of the dispersion phase, which is followed by a final elimination half-life of two hours. The dispersion phase is distinguished by its speed. There is evidence that dexmedetomidine exhibits linear kinetics at infusion rates ranging from 0.2 to 0.7 micrograms (mcg)/kg/hr for a period of up to 24 hours [6]. Fentanyl has a lot of the same effects as heroin, like euphoria, confusion, slow breathing (which can lead to cardiac arrest if it gets bad enough and isn't treated), sleeplessness, nausea, visual disturbances, dyskinesia, hallucinations, delirium (including a type of delirium called "narcotic delirium"), analgesia, constipation, narcotic ileus, muscle rigidity, addiction, loss of consciousness, low blood pressure, coma and death [7]. It may be challenging to handle multi-layered clinical circumstances when fentanyl is combined with alcohol and other drugs (such as cocaine or heroin) since these substances might intensify each other's. When combined, these chemicals cause the patient's prognosis to worsen by introducing undesirable circumstances [8]. Therefore, it is of interest to report the effective of fentanyl V/S dexmedetomidine on intubation conditions.

## Materials and Methods:

The present randomized double blind comparative study was conducted over a period of 18 months from August 2022 in the Department of Anesthesiology, Krishna Institute of Medical Sciences University and Karad with 90 patients after thorough clinical examination by trained anaesthesiologist. Here patients and researchers were blinded to group allocation with perioperative monitoring (continuous Oxygen saturation, Electrocardiogram and non-invasive blood pressure). Intravenous access was obtained with a 20G wide bore cannula on right hand through for all patients preloaded with balanced crystalloid solution (10 ml/kg) over 20 min before inducing general anesthesia with oxygen at 4L/min. Pre-medicated with tab alprazolam 0.25 mg night before surgery, inj Ranitidine 50 mg and inj Ondansetron 4 mg 1/2 h before surgery was given. Further sample was randomly divided into 2 Groups *i.e.* Group D and F with 45 each. After that dexmedetomidine (1 mcg/kg over 10 min) and fentanyl (2 mcg/kg over 10 min) was infused and evaluated by Ramsay sedation scale After this, at every 2 min, 5min, 10 min and 20 minute changes in Hemodynamic status was recorded and parameters like systolic blood pressure, diastolic blood pressure and heart rate was noted as a baseline and immediately after intubation.

## Inclusion criteria:

[1] American Society of Anesthesiologists status 1 and II

[2] Age between 20-60 years

[3] Mouth opening below 3cm/ 1 1/2 fingure

## Exclusion criteria:

[1] Pregnancy

[2] Known alcoholic / drug abuser

[3] Allergy to drugs

[4] Bradycardia

[5] Atrioventricular block

[6] Heart Failure

[7] Any disease like neurological, hepatic, renal and pulmonary.

[8] Contraindication for nasal intubation like thrombocytopenia /coagulopathy.

## Results:

Table 1 shows that, there were a total of 47 female (52.22%) patients, with 25 receiving dexmedetomidine and 22 receiving fentanyl. Among the 43 male (47.78%) patients, 20 received dexmedetomidine and 23 received fentanyl. Thus the p-value was 0.5267, which suggests that there was no statistically significant difference in the distribution. Table 2 shows that, dexmedetomidine group was 31.42 ± 7.5 years, while fentanyl group is 30.2 ± 7.16 years. Both groups have the same minimum (21 years) and maximum (48 years) ages. Thus, the p-value was 0.431 which suggests that, there was no statistically significant difference between the groups. Table 3 shows that, dexmedetomidine group was 58.82 ± 6.62 kg, whereas fentanyl group was 58.58 ± 6.28 kg. The minimum and maximum weight for the dexmedetomidine group was 48 kg and 72 kg, respectively, while for fentanyl group, they were 48 kg and 70 kg. Thus, the p-value was 0.8578 which indicates that, there was no statistically significant difference. Table 4 shows that, dexmedetomidine group was 2.820 ± 0.5599 min and standard error (SE) was 0.0835. While, at fentanyl group had 3.251 ± 0.4465 and standard error was 0.0666 respectively. Thus, the P value was less than 0.01, indicating statistically significant difference. Table 5 shows that, dexmedetomidine group was 170.89 ± 23.167 sec and standard error was 3.453. While, at fentanyl group was 188.11 ± 27.079 sec and standard error was 4.037 respectively. Thus, the P value was 0.002, indicating statistically significant difference. Table 6 shows that, dexmedetomidine group had mean cough score with 1.98 ± 1.196, while the fentanyl group had a higher mean cough score with 2.73 ± 1.053. The side effect was 0.178 for dexmedetomidine and 0.157 for fentanyl. Thus, the p-value was 0.002 indicates the statistically significant difference. Table 7 shows that at Baseline heart rate were similar as the P value was 0.57. During intubation, dexmedetomidine patients had significantly lowers heart rate than fentanyl patients were P value was 0.001. At 2 minutes, dexmedetomidine showed significantly lower heart rate as the P value was 0.001. At 4 minutes, the difference was borderline significant as the P value was 0.058 and at 6, 8, 10 and 15 minutes, the heart rate differences were not significant as the P value was > 0.05. By 20 minutes, the heart rate difference remained non-significant as the P value was 0.24. Thus results suggest that, dexmedetomidine is more effective in controlling heart rate during and shortly after intubation compared to fentanyl respectively. Table 8 shows that, Baseline systolic blood pressure was similar between the groups as the P value was 0.82. During intubation, dexmedetomidine patients had significantly lower systolic blood pressure compared to fentanyl patients as the P value was < 0.001. At 2 minutes, dexmedetomidine patients had significantly higher systolic blood pressure as the P value was 0.001 and at 4 minutes, dexmedetomidine patients had lower systolic blood pressure as the P value was 0.01. From 6 minutes onward, the differences in systolic blood pressure between the groups were not significant as the P value was > 0.05. These findings indicate that, while dexmedetomidine helps to maintain the lower systolic blood pressure during intubation, the differences become non-significant as time progresses post-intubation. Table 9 shows that, at the baseline diastolic blood pressure was similar as the P value was 0.72. During intubation, dexmedetomidine patients had significantly lower diastolic blood pressure compared to fentanyl patients as the P value was < 0.001. At 2, 4, 6, 8, 10, 15 and 20 minutes, the differences in diastolic blood pressure between the groups were not significant as the P value was > 0.05. These results suggest that, dexmedetomidine is more effective in maintaining a lower diastolic blood pressure, but the differences between the 2 groups diminish and become non-significant after intubation. Table 10 shows that, dexmed shows significantly lower mean arterial pressure during intubation (95.71 mmHg vs. 103.53 mmHg, p < 0.001) compared to Fentanyl. At other time points (2, 4, 6, 8, 10, 15 and 20 minutes), there are no significant differences in mean arterial pressure between dexmed and fentanyl groups (p > 0.05). Therefore, Dexmed appears to affect mean arterial pressure specifically during the intubation period but not significantly at other measured times. Table 11 shows that, dexmedetomidine consistently shows higher sedation score compared to fentanyl across all measured intervals: 2, 4, 6, 8 and 10 minutes (all p < 0.001). At baseline (0 minutes), there was no significant difference in sedation score between the groups as the p value was 0.32. This suggests that, dexmedetomidine induces higher levels of sedation score compared to fentanyl during the observed time points following administration. Table 12 shows that, dexmedetomidine group shows higher counts of subjects with lower tolerance scores with 66.7% scored 1, 8.9% scored 2 and 24.4% scored 3. In contrast, the fentanyl group exhibits lower counts of subjects with higher tolerance scores: 24.4% scored 1, 40.0% scored 2 and 35.6% scored 3 and thus, suggests that both groups have distinct effects on Post-intubation tolerance. Table 13 shows that, dexmedetomidine group had a lower proportion of subjects receiving rescue (6.7%) compared to fentanyl group (40.0%). Conversely, a higher proportion of dexmedetomidine subjects did not require rescue (93.3%) compared to fentanyl (60.0%). Thus, suggesting that dexmedetomidine was associated with a lower incidence of requiring rescue compared to fentanyl as the p value was < 0.01. Table 14 shows that, there were minimal occurrences of standard error overall. Specifically, O2 desaturation was reported more in the fentanyl Group (17.8%) compared to dexmedetomidine (4.4%), while erythema and flushing were rare and balanced between the Groups. Thus suggesting similar overall rates of standard error between 2 Groups as the p value was 0.15 respectively.

## Discussion:

There were 12 males and 18 females, but in Group fentanyl, there were 10 males and 20 females occupying the positions in dexmedetomidine group. The fact that the P value for the gender distribution between the two groups was 0.8663 indicates that there is no significant difference between them, which further supports the balanced allocation that was seen in this study [4]. Another study concluded that Group fentanyl had 13 males and 17 females, but Group dexmedetomidine had 8 males and 22 females, with a P value of 0.176. This finding was consistent with those of the previous study. The P value shows that this difference was not statistically significant, further supporting the assumption that gender distribution does not substantially affect the comparability of the treatment groups. This is the case, despite the fact that there seems to be a difference in the number of males and females in Group dexmedetomidine [2]. The current study shows that the mean age of the dexmedetomidine group is 31.42 ± 7.5 years, while the mean age of the fentanyl group was 30.2 ± 7.16 years. The minimum and maximum ages for both groups are the same as 21 and 48 years old, respectively. The p-value was 0.431. The results of additional investigations are in line with this trend of age similarity. Another study showed the mean ages of the dexmedetomidine and fentanyl groups were 45.10 (±3.273) and 45.57 (±3.115) years, respectively. This suggests that there was no significant age distribution difference between the groups [1]. According to a study, the intubation time for patients in Group B was 4.95 ± 3.3 seconds, which was a little less than the 55.3 ± 4.1 seconds that it took for patients in group A. The difference that was found may be attributed to the distinct mechanisms of action that are intrinsic to dexmedetomidine and propofol. These mechanisms impact the degrees of sedation in methods that are distinct from one another [9]. Our current study's enhanced timing indicates that we considered the entire duration of the endoscopy. In contrast, a study focused primarily on the intubation time [9]. This was noticed in a different experiment that was carried out by another study. They discovered that a lower loading dose of dexmedetomidine did not numb the patient enough for the first attempt at awake-fiberoptic intubation [10].

The present study revealed that, the dexmedetomidine group exhibited a mean cough score was 1.82 ±1.154 and standard error was 0.172. When compared to the other groups, individuals utilizing fentanyl exhibited a higher mean cough score was 2.31 ± 1.104 and standard error was 0.165 as the p value was 0.043. In the context of cough suppression, existing literature indicates that, dexmedetomidine demonstrates significantly greater efficacy. Although the current study did not reveal a significant difference, the existing body of evidence indicates that dexmedetomidine is typically more effective in suppressing cough than fentanyl. The differences found, how important it is to look at a lot of different studies and situations when judging how well treatments work in real life. The current investigation indicates, comparable efficacy in cough suppression between the two pharmacological agents within the defined parameters. However, the cumulative evidence tends to favor the enhanced effectiveness of dexmedetomidine in this context. This underscores the necessity for additional research and potentially more standardized methodologies to definitively address these discrepancies. Although the present study did not find any significant differences in heart rate between the dexmedetomidine and fentanyl groups, the body of evidence from other studies suggests that, dexmedetomidine often offers superior heart rate management, especially when it comes to sustaining lower heart rate levels after intubation. These differences highlight the need to take into account a variety of studies in order to gain a thorough understanding of the effectiveness and safety profiles of medications in clinical practice.

The current investigation revealed no statistically significant difference in systolic blood pressure between the groups at baseline (P = 0.82). The systolic blood pressure of patients receiving dexmedetomidine was significantly lower than that of patients receiving fentanyl during the intubation process. A statistically significant difference in systolic blood pressure was observed between individuals receiving dexmedetomidine and those not receiving at both the 2 and 4 minute marks as the p value was 0.001. The differences in systolic blood pressure among the groups were not statistically significant as the p value was 0.05 following duration of 6 minutes. The analysis showed that there was no statistically significant difference in systolic blood pressure between the fentanyl and dexmedetomidine groups at baseline as the p value was 0.64, which is similar to what Hassani *et al.* found in his 2018 study. The analysis revealed no statistically significant difference in systolic blood pressure between the groups following 5 minutes of sedation as the p value was 0.82 [11]. A trend was seen that the fentanyl group had higher systolic blood pressure (146.24 ± 30.79 mmHg) after intubation than the dexmedetomidine group (134.84 ± 12.94 mmHg), with a P value of 0.08. This suggests a potential trend, although it does not reach statistical significance. The current study indicates that, dexmedetomidine considerably reduces mean arterial pressure during intubation (95.71 mmHg vs. 103.53 mmHg, p < 0.001) in comparison to fentanyl. At further time intervals (2, 4, 6, 8, 10, 15 and 20 minutes), no significant differences in mean arterial pressure were seen between the dexmedetomidine and fentanyl groups (p >0.05). Previous studies reveal substantial discrepancies in mean arterial pressure between the dexmedetomidine and fentanyl groups under different settings. In clinical practice, comprehending these distinctions is essential for choosing the suitable sedative according to the intended CV effects. Dexmedetomidine capacity to provide hemodynamic stability, particularly by sustaining lower mean arterial pressure, may be beneficial in situations requiring meticulous management of cardiovascular function, such as during intubation operations. Nonetheless, unique patient characteristics and particular clinical circumstances have to inform therapy choices to enhance results and ensure patient safety. In a similar study reported a significantly elevated Ramsay sedation scale in Group dexmedetomidine 3 ± 0.371) when compared to Group F (2.1 ± 0.254) at the conclusion of the drug infusion (P < 0.0001). The results of this investigation are consistent with the significant finding observed at the 8-minute mark, indicating that; dexmedetomidine produced superior sedation score in comparison to Fentanyl. The observed differences in sedation efficacy between dexmedetomidine and fentanyl across various studies can be attributed to multiple factors, including the pharmacokinetics and pharmacodynamics of the medications, dosing regimens, patient populations and the specific sedation assessment tools employed, such as the Ramsay sedation scale [12]. Dexmedetomidine often provides superior intubation conditions compared to fentanyl, according to the significant differences reported in these studies.

The recurrent observation of significantly improved post intubation score with dexmedetomidine in several studies demonstrates its potential benefit in aiding ideal intubation conditions. Variations in study design, patient demographic characteristics, or intubation grading criteria could be the cause of the inconsistencies in the current study's results. The findings of the current study show that the scores obtained by the two groups are quite similar, suggesting that both drugs have equivalent effectiveness within the current studies stated conditions. Nonetheless, the body of evidence suggests a trend in favour of using dexmedetomidine for improved intubation conditions. This disparity highlights the need to consider a wide range of studies and circumstances when assessing clinical outcomes and determining treatment choices. Dexmedetomidine was shown to give superior hemodynamic stability and deeper sedation levels, requiring significantly less rescue medications than Fentanyl. Because of its alpha-2 adrenergic agonist characteristics, these studies together suggest that dexmedetomidine provides more consistent and sustained sedation, reducing the chance of requiring RM during operations [13]. In a similar vein, a study observed a markedly reduced incidence of desaturation in the dexmedetomidine cohort (4 patients) when juxtaposed with the fentanyl cohort (25 patients), achieving a highly significant p-value (P <0.0001) [14]. Furthermore, study reported a reduced occurrence of desaturation associated with the use of dexmedetomidine in comparison to remifentanil study showed that dexmedetomidine provides optimum sedation without compromising airway or hemodynamic stability and with favorable intubation time and less intubation attempts during Awake-fibreoptic intubation in comparison to fentanyl [15]. The results of another study showed that dexmedetomidine provides optimum sedation without compromising airway or hemodynamic stability and with favorable intubation time and less intubation attempts during awake-fibreoptic intubation in comparison to fentanyl [16]. Another study showed that, dexmedetomidine was found to be more effective than clonidine and fentanyl in that undergoing awake-fibreoptic intubation. There were fewer adverse effects such as coughing, discomfort, oxygen desaturation and intolerance to intubation [17].

## Conclusion:

Dexmedetomidine worked better than fentanyl at putting people to sleep, stopping coughing and keeping their blood pressure stable for awake-fibreoptic intubation procedure. The outcomes included a notable reduction in cases necessitating rescue medications, enhanced sedation score and improved tolerance to intubation. Both Groups reported minimal standard error, with dexmedetomidine showing a reduced incidence of O2 desaturation. In comparison to Fentanyl, dexmedetomidine offers a safer and more effective sedation profile, accompanied by a reduced incidence of standard error.

## Figures and Tables

**Table 1:** Sex distribution

**SEX**	**Group**		**Total**	**p-value**
	**Dexmedetomidine**	**Fentanyl**		
Female	25	22	47	0.5267
	27.78	24.4	52.22	
Male	20	23	43	
	22.22	25.6	47.78	

**Table 2:** Age distribution

**Variable**	**Label**	**N**	**Mean**	**Std. Dev(SD)**	**Minimum**	**Maximum**	**p-value**
DEX	AGE	45	31.42	7.5	21	48	0.431
Fentanyl	AGE	45	30.2	7.16	21	48	

**Table 3:** Weight distribution

**Variable**	**N**	**Mean**	**Std. Dev**	**Minimum**	**Maximum**	**p-value**
DEX	45	58.82	6.62	48	72	0.8578
Fentanyl	45	58.58	6.28	48	70	

**Table 4:** Mean Endoscopy time

	**Group**	**N**	**Mean**	**Std. Dev**	**Std. Error Mean**	**P value**
Endoscopy time (ED-T)	Dexmedetomidine	45	2.820( 2 min 49 sec)	0.5599	0.0835	<0.01,
	Fentanyl	45	3.251(3 min 15 sec)	0.4465	0.0666	significant

**Table 5:** Mot distribution

	**Group**	**N**	**Mean**	**Std. Dev**	**SE**	**P-value**
MEAN ONSET TIME (MOT) (sec)	DEX	45	170.89( 2 min 51 sec)	23.167	3.453	0.002
	Fentanyl	45	188.11(3 minutes 8 sec)	27.079	4.037	

**Table 6:** Cough Score distribution

	**Group**	**N**	**Mean**	**Std. Dev**	**SE**	**P-value**
Cough Score	DEX	45	1.98	1.196	0.178	0.002,
(CS)	Fentanyl	45	2.73	1.053	0.157	significant

**Table 7:** M-Heart rate distribution

	**Group**	**N**	**Mean**	**Std. Dev**	**P-value**
Baseline (BS)	DEX	45	80.64	8.671	0.57
HR	Fentanyl	45	81.67	8.658	
HR during IT	DEX	45	86.64	8.671	<0.001
	Fentanyl	45	95.07	8.695	
HR at 2 min	DEX	45	82.11	7.802	0.001
	Fentanyl	45	88.07	8.695	
HR at 4 min	DEX	45	82.42	9.631	0.058
	Fentanyl	45	85.8	6.818	
HR at 6 min	DEX	45	83.51	7.494	0.13
	Fentanyl	45	81.18	7.136	
HR at 8 min	DEX	45	81.73	6.933	0.09
	Fentanyl	45	79.2	7.083	
HR at10 min	DEX	45	82.64	7.935	0.9
	Fentanyl	45	82.53	8.379	
HR at 15 min	DEX	45	81.13	6.28	0.7
	Fentanyl	45	80.62	6.648	
HR at 20 min	DEX	45	81.69	7.695	0.24
	Fentanyl	45	83.51	7.789	

**Table 8:** M-systolic blood pressure distribution

	**Group**	**N**	**Mean**	**Std. Dev**	**P-value**
baseline SBP	Dexmedetomidine	45	118.49	6.781	0.82
	Fentanyl	45	118.78	5.563	
SBP during intubation	Dexmedetomidine	45	128.49	6.781	<0.001
	Fentanyl	45	134.78	5.563	
SBP at 2 min	Dexmedetomidine	45	122.31	6.41	0.001
	Fentanyl	45	117.93	5.272	
SBP at 4 min	Dexmedetomidine	45	123.33	7.447	0.01
	Fentanyl	45	126.47	3.811	
SBP at 6 min	Dexmedetomidine	45	119.13	4.566	0.9
	Fentanyl	45	119.24	4.211	
SBP at 8 min	Dexmedetomidine	45	118.36	5.219	0.33
	Fentanyl	45	119.44	5.413	
SBP at 10 min	Dexmedetomidine	45	119.31	4.374	0.75
	Fentanyl	45	119.04	3.808	
SBP at 15 min	Dexmedetomidine	45	118.09	5.942	0.5
	Fentanyl	45	118.98	6.552	
SBP at 20 min	Dexmedetomidine	45	119.18	4.469	0.49
	Fentanyl	45	118.58	3.769	

**Table 9:** M-diastolic blood pressure distribution

	**Group**	**N**	**Mean**	**Std. Dev**	**P-value**
baseline DIASTOLIC BLOOD PRESSURE	Dexmedetomidine	45	75.62	5.149	0.72
	Fentanyl	45	75.27	4.234	
DIASTOLIC BLOOD PRESSURE during intubation	Dexmedetomidine	45	81.62	5.149	<0.001
	Fentanyl	45	86.27	4.234	
DIASTOLIC BLOOD PRESSURE at 2 min	Dexmedetomidine	45	75.29	4.65	0.43
	Fentanyl	45	74.58	3.829	
DIASTOLIC BLOOD PRESSURE at 4 min	Dexmedetomidine	45	81.31	5.008	0.25
	Fentanyl	45	80.18	4.433	
DIASTOLIC BLOOD PRESSURE at 6 min	Dexmedetomidine	45	75.93	4.721	0.62
	Fentanyl	45	75.44	4.77	
DIASTOLIC BLOOD PRESSURE at 8 min	Dexmedetomidine	45	75.58	4.003	0.67
	Fentanyl	45	75.96	4.462	
DIASTOLIC BLOOD PRESSURE at 10 min	Dexmedetomidine	45	76.27	4.942	0.97
	Fentanyl	45	76.22	4.972	
DIASTOLIC BLOOD PRESSURE at 15 min	Dexmedetomidine	45	74.67	4.467	0.57
	Fentanyl	45	75.22	4.885	
DIASTOLIC BLOOD PRESSURE at 20 min	Dexmedetomidine	45	74.38	4.075	0.18
	Fentanyl	45	75.58	4.382	

**Table 10:** M- Mean arterial pressure distribution

	**Group**	**N**	**Mean**	**Std. Dev**	**P-value**
baseline	Dexmedetomidine	45	89.62	5.399	0.93
MAP	Fentanyl	45	89.53	4.257	
MAP during intubation	Dexmedetomidine	45	95.71	4.93	<0.001
	Fentanyl	45	103.53	4.257	
MAP	Dexmedetomidine	45	89.47	4.516	0.41
at 2 min	Fentanyl	45	88.76	3.675	
MAP	Dexmedetomidine	45	96.09	4.161	0.31
at 4 min	Fentanyl	45	95.27	3.57	
MAP	Dexmedetomidine	45	90	3.778	0.76
at 6 min	Fentanyl	45	89.76	3.85	
MAP	Dexmedetomidine	45	89.7333	3.78033	0.38
at 8min	Fentanyl	45	89.0222	3.91088	
MAP	Dexmedetomidine	45	89.49	3.565	0.43
at 10 min	Fentanyl	45	90.11	4.013	
MAP	Dexmedetomidine	45	88.84	4.067	0.49
at 15 min	Fentanyl	45	89.47	4.615	
MAP	Dexmedetomidine	45	89	3.405	0.41
at 20 min	Fentanyl	45	89.58	3.173	

**Table 11:** M-sedation score distribution

	**Group**	**N**	**Mean**	**Std. Dev**	**P-value**
sedation score(SS)	Dexmedetomidine	45	1	0	0.32
at 0 min	Fentanyl	45	1.02	0.149	
SS at 2 min	Dexmedetomidine	45	2.02	0.941	<0.001
	Fentanyl	45	1.16	0.475	
SS at 4 min	Dexmedetomidine	45	2.04	0.903	<0.001
	Fentanyl	45	1.22	0.56	
SS at 6 min	Dexmedetomidine	45	2.93	0.252	<0.001
	Fentanyl	45	2.07	0.863	
SS at 8 min	Dexmedetomidine	45	2.93	0.495	<0.001
	Fentanyl	45	2.13	0.625	
SS at 10 min	Dexmedetomidine	45	3.2	0.548	<0.001
	Fentanyl	45	2.93	0.252	

**Table 12:** Post Intubation Score distribution

			**Group**		**Total**
			**Dexmedetomidine**	**Fentanyl**	
Post Intubation Score (P-IT-S)	1	Count	30	11	41
		% within group	66.70%	24.40%	45.60%
	2	Count	4	18	22
		% within group	8.90%	40.00%	24.40%
	3	Count	11	16	27
		% within group	24.40%	35.60%	30.00%
Total		Count	45	45	90
		% within group	100.00%	100.00%	100.00%
Chi-sq value-18.64, p value- <0.001, significant					

**Table 13:** Rescue distribution

			**Group**		**Total**
			**Dexmedetomidine**	**Fentanyl**	
Rescue	No	Count	42	27	69
		% within group	93.30%	60.00%	76.70%
	yes	Count	3	18	21
		% within group	6.70%	40.00%	23.30%
Total		Count	45	45	90
		% within group	100.00%	100.00%	100.00%
Chi-sq value-13.97, p value- <0.01, significant					

**Table 14:** Side effects distribution

			**Group**		**Total**
			**Dexmedetomidine**	**Fentanyl**	
Side Effects	Erythema	Count	0	1	1
Side Effects		% within group	0.00%	2.20%	1.10%
	Flushing	Count	1	1	2
		% within group	2.20%	2.20%	2.20%
	O2 Desaturation	Count	2	8	10
		% within group	4.40%	17.80%	11.10%
	No	Count	42	35	77
		% within group	93.30%	77.80%	85.60%
Total		Count	45	45	90
		% within group	100.00%	100.00%	100.00%
Chi-sq value-0.23, p value-0.15, non-significant					
